# Diagnosis, testing, treatment, and outcomes among patients with advanced non‐small cell lung cancer in the United States

**DOI:** 10.1002/cam4.6694

**Published:** 2023-12-07

**Authors:** Mo Yang, Joanna P. MacEwan, Sai Sriteja Boppudi, Monica R. McClain, Richard M. O'Hara, Paul K. Paik

**Affiliations:** ^1^ EMD Serono Rockland Massachusetts USA; ^2^ Genesis Research Hoboken New Jersey USA; ^3^ Memorial Sloan Kettering Cancer Center New York New York USA

**Keywords:** advanced NSCLC, biomarker testing, outcomes, real‐world data, targeted therapy

## Abstract

**Introduction:**

Characteristics of patients in clinical trials may differ from those of real‐world patients. Our objective was to describe biomarker testing and outcomes among patients with advanced non‐small cell lung cancer (aNSCLC) in a real‐world setting.

**Methods:**

This retrospective cohort study included patients ≥18 years old, diagnosed with stage IIIB/C or IV NSCLC, and in the TEMPUS oncology dataset from January 1, 2012, to December 31, 2020. Patient characteristics associated with biomarker testing were evaluated in patients with positive biomarkers using univariate logistic regression models. Cox proportional hazard models were used to estimate median survival.

**Results:**

Of 9540 patients included, 41.7% had biomarker testing, and 2158 had a positive biomarker result. Men (vs women; odds ratio [OR], 0.82; 95% CI: 0.74–0.91), Black patients (vs White; OR, 0.83; 95% CI: 0.72–0.97), patients with squamous (OR, 0.22; 95% CI: 0.19–0.25) or unknown histology (OR, 0.53; 95% CI: 0.45–0.61) (vs non‐squamous histology), and patients with an Eastern Cooperative Oncology Group performance status (ECOG PS) of 2+ (OR, 0.69; 95% CI: 0.57–0.84) or missing (OR, 0.56; 95% CI: 0.48–0.66) (vs ECOG PS of 0) were less likely to undergo biomarker testing. Patients with positive biomarkers who received NCCN‐recommended treatment options (55.7%) had significantly longer median overall survival (OS) (hazard ratio [HR], 0.84; 95% CI: 0.75–0.95) and real‐world progression‐free survival (rwPFS) (HR, 0.68; 95% CI: 0.62–0.75).

**Conclusion:**

More than 50% of patients were untested for biomarkers. Patients who were less likely to be tested included men, Black patients, current smokers, patients with squamous aNSCLC, and patients with an ECOG PS of 2+. Patients with positive biomarkers who received NCCN‐recommended treatment options had significantly longer OS and PFS.

## Graphical abstract

Despite new targeted therapies for advanced NSCLC (aNSCLC) and improvements in testing, our study found that more than half of patients with aNSCLC in the TEMPUS oncology dataset were untested for biomarkers and patient groups that were least likely to be tested included men, Black patients, current smokers, patients with squamous aNSCLC, and patients with an Eastern Cooperative Oncology Group performance status (ECOG PS) of 2 or more. In this real‐world setting, patients with aNSCLC who were tested for biomarkers and received an NCCN‐recommended treatment had longer median overall survival and real‐world progression‐free survival compared with patients treated with non‐recommended regimens.

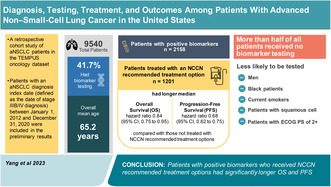

## 1 INTRODUCTION

In the United States (US), lung cancer is the leading cause of cancer deaths and the third most common cancer type; estimates project approximately 283,340 new cases in 2023.^1^ It is also the leading cause of cancer deaths in the US, with a projected 127,070 deaths for 2023.^1^ Non‐small cell lung cancer (NSCLC) is the most common form of lung cancer, making up approximately 84% of lung cancer cases from 2010 to 2017.^2^ Approximately two‐thirds of patients with NSCLC are diagnosed at stage III or IV.^3^, ^4^ For patients with stage IV NSCLC, the 5‐year survival rate has been estimated to be as low as 6%.^2^

Testing to define molecular profiles and immunologic status can help individualize treatment for patients with advanced NSCLC (aNSCLC) and determine the most appropriate treatment options. According to the National Comprehensive Cancer Network Clinical Practice Guidelines in Oncology (NCCN Guidelines^®^) NSCLC version 5.2021, patients with aNSCLC who harbor *ALK*, *ROS1*, or *RET* rearrangements, *EGFR*, *MET*, *BRAF*, or *KRAS* mutations, *NTRK* fusions, and/or a high programmed death‐ligand 1 (PD‐L1) level should have US Food and Drug Administration (FDA)‐approved targeted treatment.^5^ With the introduction of targeted therapies, such as *EGFR* and *ALK* inhibitors, mortality from NSCLC has decreased more quickly than incidence from 2013 through 2016.^6^ Recently, gene alterations such as *MET* exon 14 skipping and *HER2* mutations have been shown to be promising targets for treatments.^7^, ^8^, ^9^, ^10^

Despite recent developments in testing techniques and treatments, a need to better understand diagnosis and testing as well as treatment patterns and outcomes for patients with aNSCLC outside of the clinical trial setting remains. Our objective was to describe the demographics and clinical characteristics, biomarker testing and treatment patterns, and survival outcomes in patients with aNSCLC in a real‐world database.

## 2 METHODS

### 2.1 Study design and database description

This was a retrospective US cohort study. Data were collected from the TEMPUS oncology real‐world dataset. The observation period was from January 1, 2012, to December 31, 2020. The TEMPUS oncology real‐world dataset consists of 3.8 million de‐identified records. Data are collected from oncology practices across the US. Data from electronic health record systems are integrated with other available patient data, including biomarker data, via technology‐enabled chart abstraction and biomarker data.

Data were organized into three modalities. Clinical‐only (CO) modality records were abstracted from unstructured sources along with structured data and received through TEMPUS' CancerLinQ (CLQ) partnership. These records do not contain TEMPUS sequenced biomarker data. The clinical‐genomic (CG) modality contained clinical data from structured electronic health record (EHR) sources along with abstracted unstructured sources through the TEMPUS curation pipeline. In addition, biomarker data from TEMPUS' in‐house sequencing were provided through group‐level biomarker data. The multimodal (MM) database contained the same data as the CG modality, as well as biomarker data provided through individual patient‐level files.

The TEMPUS dataset contains de‐identified data; therefore, no institutional review board or ethics committee approval was required.

### 2.2 Participants

This study included adult patients, aged 18 years and older, diagnosed with aNSCLC between January 1, 2012, and December 31, 2020. Criteria for aNSCLC included evidence of stage IIIB–C or IV disease at any time, or an associated metastatic event. The index date was defined as the earliest date of stage IIIB–C/IV or metastatic diagnosis within the study period. Patients were excluded from the study if age or sex information was missing, histology results suggested a different diagnosis than NSCLC, or death occurred prior to other events.

### 2.3 Variables and outcomes

Baseline demographics and clinical characteristics included sex (male or female); age at index; age group at index (≤50, 51 to 64, ≥65 years); race (Black/African American, White, other Race including American Indian or Alaska Native, Asian, Native Hawaiian or Other Pacific Islander, other race, or unknown); smoking status (current smoker, former smoker, never smoked tobacco, or unknown); stage at index (IIIB–C or IV); Eastern Cooperative Oncology Group performance status (ECOG PS) (0, 1, 2+, or missing); presence of brain metastasis on or before diagnosis, where noted; and histology at index (non‐squamous, other/unknown, squamous, or missing).

The presence or absence and result of documented biomarker testing for *ALK*, *ROS1*, or *RET* rearrangements, *EGFR*, *MET*, *BRAF*, or *KRAS* mutations, *NTRK* fusions, and/or PD‐L1 were assessed. If multiple testing results were recorded, a positive test was prioritized over a negative one and a negative test over an unknown. Patient characteristics, including sex, race, smoking status, ECOG PS, histology, and timing associated with biomarker testing were evaluated.

Treatment patterns and duration of treatment (DoT) were assessed by line of treatment (LoT) and drug class. DoT was defined as the duration between the date of treatment initiation and the first occurrence of any of the following: the last infusion or fill date of all drugs in the LoT regimen, the start of the next LoT, or death.

If a patient was identified as having a gene alteration, and/or PD‐L1 ≥ 1%, evaluation was performed regarding the use of NCCN Guidelines^®^ ^5^ NSCLC version 5.2021 for targeted treatment or immunotherapy options in any LoT. Clinical outcomes were stratified by patients with positive biomarkers who received NCCN‐recommended treatment options compared with those who did not.

Clinical outcomes included median overall survival (OS) and median real‐world progression‐free survival (rwPFS). OS was defined as the duration between index date and the end of follow‐up (the date of death, date of last contact, or the end‐of‐study follow‐up period—whichever occurred first). Real‐world PFS was defined as the duration between index date and date of initiation of second‐line treatment post index, documented progression, date of death, or the end‐of‐study follow‐up period—whichever occurred first.

As data from the MM and CG TEMPUS modalities only contain patients who received biomarker testing, results for variables assessing counts, rates, and patterns of biomarker testing and patient characteristics associated with specific biomarker testing were obtained from the CO modality only.

### 2.4 Statistical analysis

As this was an exploratory study, there were no formal calculations of sample size and statistical power. Descriptive statistics were used to summarize patient demographics and clinical characteristics, biomarker results, and treatment patterns. Among patients from the CO modality, univariate logistic regression models were used to evaluate the factors associated with biomarker testing. Odds ratios (ORs) with 95% confidence intervals (CIs) and *p* values were reported. Among patients who tested positive for at least one biomarker, a Cox proportional hazards model estimated median OS and rwPFS, stratified by receipt of NCCN‐recommended treatment in any LoT and adjusted for demographic and clinical characteristics at baseline including sex, age group at diagnosis, race, smoking status, ECOG PS, and histology. Hazard ratios (HRs) with 95% CIs were reported. Immortal time bias correction was used to account for the requirement that patients survive long enough to be tested. In all analyses, missing data were considered a separate category and were described using frequency counts and percentages. Missing data were not imputed. All tests were conducted with pre‐specified critical *p* values < 0.05 as the threshold for statistical significance. All data management tasks and analyses were conducted using R version 3.6.0.

## 3 RESULTS

### 3.1 Demographics and baseline characteristics

**FIGURE 1:**
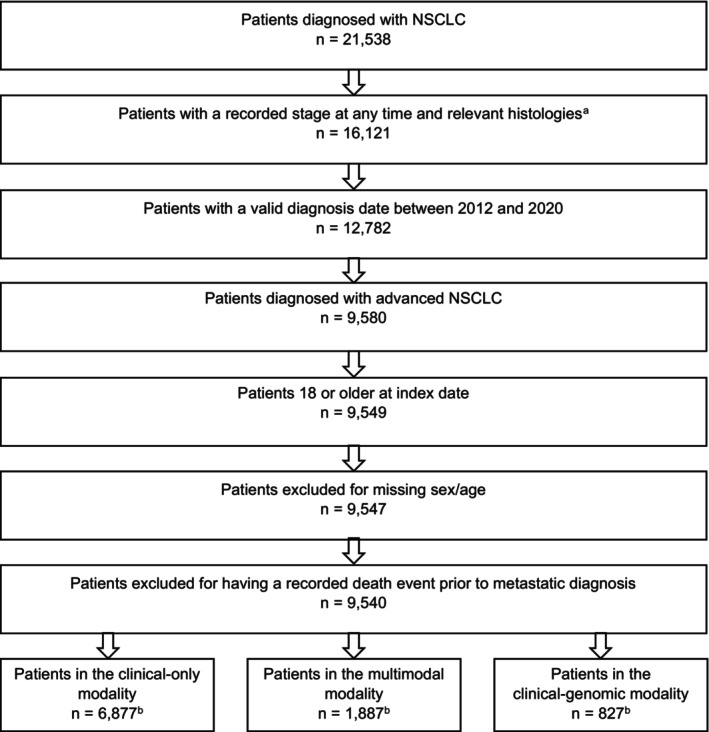
Patient Attrition. NSCLC, non‐small cell lung cancer. ^a^Patients with histologies that were not consistent with NSCLC were excluded. ^b^Numbers do not total 9540 because 51 patients were duplicated in the clinical‐only and clinical‐genomic/multimodal databases.

Among 21,538 patients diagnosed with NSCLC recorded in the TEMPUS dataset, 9549 patients met the inclusion criteria. Two patients were excluded due to missing sex/age information and seven due to deaths occurring prior to other events, leaving 9540 patients remaining (Figure 1). There were 6877 patients (72.1%) in the CO modality, 1887 (19.8%) in the MM modality, and 827 (8.7%) in the CG modality. Numbers do not total 9540 because 51 patients were duplicated in the CO or MM/CG modality. There were 2158 patients who tested positive for at least one biomarker.

Patient demographics and clinical characteristics are summarized in Table 1. The overall population had an even sex distribution, a mean age of 65.2 years, and a majority of patients were White (68.6%). Approximately half (47.9%) of the overall population included either current or former smokers, although 42.0% had an unknown smoking status. Most patients were diagnosed at stage IV (90.0%) and had an ECOG PS ≤1 (44.7%), although 40.3% were missing an ECOG PS. There were 1102 patients (11.5%) with evidence of brain metastasis on or before diagnosis. Most patients had non‐squamous histology (64.6%). No notable differences were found between patient characteristics stratified by data modality.

**TABLE 1:** Patient characteristics for overall cohort, clinical‐only, and biomarker‐positive patients with advanced non‐small cell lung cancer^a^ ^,^ ^b^.

Characteristic	Overall *n* = 9,591^c^	Clinical‐only *n* = 6877	Biomarker positive^d^ *n* = 2158
Sex			
Female	4565 (47.6)	3168 (46.1)	1181 (54.7)
Male	5026 (52.4)	3709 (53.9)	977 (45.3)
Age at index, years			
Mean (SD)	65.2 (9.9)	65.2 (9.8)	65.3 (10.2)
Median (IQR)	65.6 (58.6–72.7)	65.6 (58.5–72.7)	65.6 (58.8–72.9)
Range	20.2–87.1	23.9–87.1	25.6–87.1
Age group at index, years			
≤50	654 (6.8)	445 (6.5)	157 (7.3)
51–64	3786 (39.5)	2730 (39.7)	841 (39.0)
≥65	5046 (52.6)	3626 (52.7)	1140 (52.8)
Missing or unknown	105 (1.1)	76 (1.1)	20 (0.9)
Race			
White	6577 (68.6)	5027 (73.1)	1355 (62.8)
Black or African American	1166 (12.2)	917 (13.3)	209 (9.7)
Other^e^	695 (7.2)	452 (6.6)	222 (10.3)
Missing or unknown	1153 (12.0)	481 (7.0)	372 (17.2)
Smoking status			
Current smoker	1904 (19.9)	1405 (20.4)	336 (15.6)
Former smoker	2684 (28.0)	1717 (25.0)	668 (31.0)
Never smoked tobacco	979 (10.2)	459 (6.7)	403 (18.7)
Missing or unknown	4024 (42.0)	3296 (47.9)	751 (34.8)
Stage at index			
IIIB	887 (9.2)	623 (9.1)	157 (7.3)
IIIC	70 (0.7)	31 (0.5)	11 (0.5)
IV	8634 (90.0)	6223 (90.5)	1990 (92.2)
ECOG PS			
0	1602 (16.7)	941 (13.7)	496 (23.0)
1	2685 (28.0)	1628 (23.7)	727 (33.7)
2	1073 (11.2)	770 (11.2)	223 (10.3)
3	314 (3.3)	245 (3.6)	57 (2.6)
4	52 (0.5)	46 (0.7)	14 (0.6)
Missing	3865 (40.3)	3247 (47.2)	641 (29.7)
Brain metastasis^f^	1102 (11.5)	751 (10.9)	244 (11.3)
Histology at index			
Non‐squamous	6195 (64.6)	4240 (61.7)	1725 (79.9)
Other/unknown	1164 (12.1)	875 (12.7)	206 (9.5)
Squamous	2229 (23.2)	1762 (25.6)	225 (10.4)
Missing	3 (0.0)	0 (0.0)	2 (0.1)

Abbreviations: ECOG PS, Eastern Cooperative Oncology Group performance status; IQR, interquartile range; SD, standard deviation.

^a^ Expressed as No. (%) unless otherwise indicated.

^b^ Percentages may not equal 100% due to rounding.

^c^ Fifty‐one patients were found to have duplicated records in the clinical‐only and clinical‐genomic/multimodal databases.

^d^ Positive for at least one biomarker.

^e^ Small sample size did not allow for disaggregation of the Other Race category.

^f^ Excludes brain metastasis recorded after aNSCLC diagnosis date.

### 3.2 Patient characteristics associated with biomarker testing

There were 2869 patients (41.7%) in the CO modality who received biomarker testing. The most common biomarker tested was *EGFR* (53.3%) followed by *ALK* (42.3%). The least common was *NTRK* (1.1%).

Men were significantly less likely to undergo biomarker testing compared with women (OR, 0.82; 95% CI: 0.74–0.91). Compared with White patients, Black patients were less likely to receive biomarker testing (OR, 0.83; 95% CI: 0.72–0.97). Patients with squamous histology and patients with unknown histology were less likely to undergo biomarker testing than patients with non‐squamous histology (OR, 0.22; 95% CI: 0.19–0.25 and OR, 0.53; 95% CI: 0.45–0.61). Patients with an ECOG PS of 2 or more and patients missing an ECOG PS score were less likely to receive biomarker testing than patients with an ECOG PS of 0 (OR, 0.69; 95% CI: 0.57–0.84 and OR, 0.56; 95% CI: 0.48–0.66). Never smokers were more likely to receive biomarker testing than current smokers (OR, 2.64; 95% CI: 2.05–3.42). Patients diagnosed each year from 2016 to 2020 were more likely to undergo biomarker testing than patients diagnosed in 2012 (Table 2).

**TABLE 2:** Patient characteristics associated with biomarker testing in patients with advanced non‐small cell lung cancer.

Variable	Odds ratio	95% CI lower border	95% CI upper border	*p* value
Intercept	2.04	1.52	2.76	<0.001
Sex, male	0.82	0.74	0.91	<0.001
Age group at diagnosis				
51–64 years	0.78	0.62	0.97	0.030
≥65 years	0.69	0.55	0.86	0.001
Race				
Black or African American	0.83	0.72	0.97	0.020
Other	1.44	1.16	1.79	0.001
Unknown	1.03	0.84	1.27	0.768
Smoking status				
Former smoker	1.49	1.27	1.74	<0.001
Never smoked tobacco	2.64	2.05	3.42	<0.001
Unknown	1.35	1.17	1.55	<0.001
ECOG PS				
1	0.93	0.78	1.11	0.414
2+	0.69	0.57	0.84	<0.001
Missing	0.56	0.48	0.66	<0.001
Histology				
Other/Unknown	0.53	0.45	0.61	<0.001
Squamous	0.22	0.19	0.25	<0.001
Year of advanced diagnosis				
2013	1.09	0.89	1.34	0.398
2014	1.23	1.01	1.51	0.039
2015	1.21	1.00	1.47	0.055
2016	1.50	1.23	1.83	<0.001
2017	2.77	2.25	3.41	<0.001
2018	2.48	2.00	3.07	<0.001
2019	1.74	1.34	2.26	<0.001
2020	2.07	1.40	3.09	<0.001

*Note*: Reference groups include females, age ≤ 50 years, White race, current smoker, ECOG PS of 0, non‐squamous histology, and diagnosis year of 2012.

Abbreviations: CI, confidence interval; ECOG PS, Eastern Cooperative Oncology Group performance status.

### 3.3 Treatment patterns

During the study period, chemotherapy was the most common class of treatment in all LoTs. Chemotherapy plus checkpoint inhibitor (CPI) and CPI alone were the second most common treatments in first line (1L) and second/third line (2L/3L), respectively. Of the 9540 patients, there were 6391 patients who received 1L treatment; of those, 56.3% were treated with chemotherapy and 19.8% were treated with chemotherapy and CPI. Of the 6391 patients, 43.4% (2771) advanced to 2L. Of those 2771 patients, 43.4% received chemotherapy and 32.8% CPI alone. Of the 6391 patients, 17.0% (1085) advanced to 3L. Of those 1085, 51.4% received chemotherapy and 23.4% CPI alone (Table S1).

From 2016 through 2020, 4410 patients received 1L treatment, and of those 4410 patients, chemotherapy was most common, at 37.4%. Of the 4410 patients, 37.5% (1655) advanced to 2L, and of those 1655 patients, CPI was most common at 40.2%. Of the 4410 patients, 13.3% (586) received 3L treatment. Of the patients in 1L (4,410) and 2L (1,655), most received chemotherapy plus CPI, CPI alone, or tyrosine kinase inhibitor (TKI): 61.3% and 64.2%, respectively (Table S1).

### 3.4 Clinical outcomes stratified by NCCN‐recommended treatment options

**FIGURE 2:**
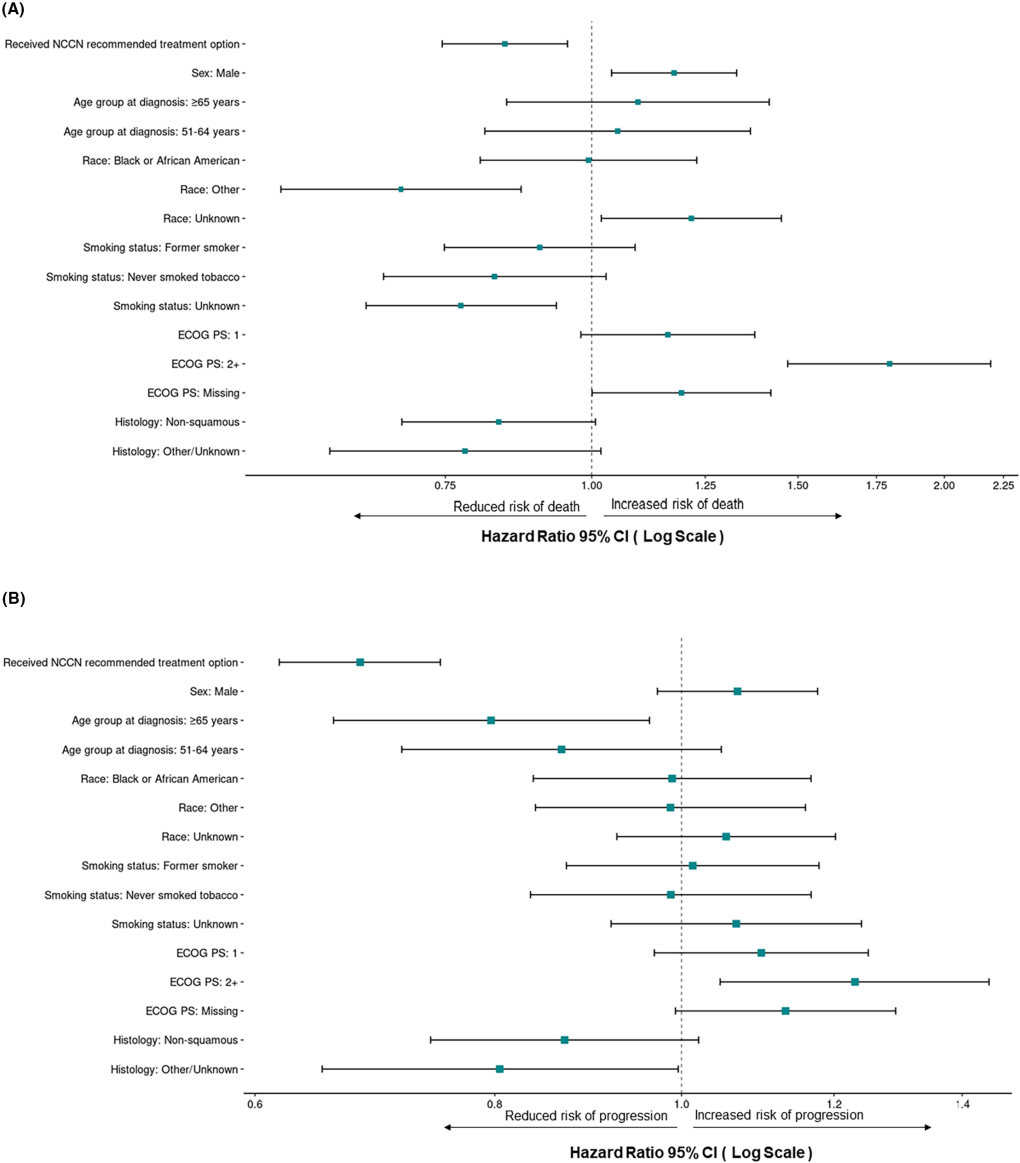
Survival by NCCN treatment status among biomarker‐positive patients with advanced non‐small cell lung cancer. (A) Median overall survival and (B) median real‐world progression‐free survival by NCCN‐recommended treatment status. The Other race category included American Indian or Alaska Native, Asian, Native Hawaiian or Other Pacific Islander, and other race. Reference groups include: no NCCN‐recommended treatment, females, age ≤ 50 years, White race, current smoker, ECOG PS of 0, and squamous histology. CI, confidence interval; ECOG PS, Eastern Cooperative Oncology Group performance status; NCCN, National Comprehensive Cancer Network.

Of the 2158 patients with positive biomarkers, PD‐L1 ≥ 1% (38.0%), *EGFR* (32.2%), and/or *KRAS* (30.3%) were the most common gene alterations, and 1201 (55.7%) patients received an NCCN‐recommended treatment option. These patients had significantly longer median OS (HR, 0.84; 95% CI: 0.75−0.95) compared with those not treated with NCCN‐recommended treatment options (Figure 2A). Furthermore, patients with positive biomarkers who received NCCN‐recommended treatment had significantly longer median rwPFS (*n* = 1199; HR, 0.68; 95% CI: 0.62−0.75) (Figure 2B). These results were qualitatively unchanged when patients with *KRAS* mutations, for which there were no approved therapies available during the study period, were excluded.

Several patient characteristics were also associated with significant differences in OS and rwPFS (Figure 2A,B). Patients with an unknown smoking history had a longer median OS compared with current smokers (HR, 0.77; 95% CI: 0.64−0.93). Men (compared with women) had a shorter median OS (HR, 1.18; 95% CI: 1.04−1.33) along with patients with an ECOG PS of 2+ (HR, 1.80; 95% CI: 1.47−2.19) compared with an ECOG PS of 0. Patients within the Other Race category (compared with White patients) had a longer median OS (HR, 0.69; 95% CI: 0.54−0.87). When assessing median rwPFS, patients with an ECOG PS of 2+ (compared with ECOG PS of 0) had a shorter median rwPFS (HR, 1.23; 95% CI: 1.05−1.45). There were no significant differences in median rwPFS based on smoking status, sex, or race.

## 4 DISCUSSION

Despite the increasing availability of targeted therapies for aNSCLC and the relative ease of currently available biomarker tests, many patients in our study did not receive testing. Only 41.7% of eligible patients received biomarker testing. Another real‐world study of patients with aNSCLC between 2011 and 2019 found slightly higher rates of biomarker testing: 68.7% of patients.^11^ The higher rates in the John et al. study could be due to the use of different databases or observational periods or both. Real‐world studies with more recent observational periods and different databases than our study have reported higher biomarker testing rates: approximately 90% of patients with at least one biomarker result available.^12^, ^13^ In our study, patients diagnosed after 2015 were more likely to be tested than those in 2012. As many of the biomarker‐targeted treatments were approved after 2016,^14^ changes in the diagnosis and management of aNSCLC during the observational period of our study could explain the lower rates of testing we found compared with the aforementioned studies.

Another similar study found higher biomarker testing rates for *EGFR*, *ALK*, *ROS1*, and *BRAF* in patients with non‐squamous NSCLC compared with their overall population of patients with metastatic NSCLC.^13^ In our study, we found that patients with squamous aNSCLC were significantly less likely to undergo biomarker testing compared with patients with non‐squamous histology. In light of the lack of actionable biomarkers specific to squamous aNSCLC and the recommendations to test only squamous aNSCLC in never smokers and those diagnosed through needle biopsy that were in place until 2020, this is not surprising.^15^ The lack of testing in squamous aNSCLC is problematic, given the occurrence of several actionable alterations such as *EGFR* and *MET* that can occur,^16^ albeit at low frequency. Subsequent revisions of the NCCN guidelines (2020) included recommendations to consider routine comprehensive molecular testing for patients with squamous aNSCLC.^5^

*EGFR* and *ALK* were the most commonly tested biomarkers in our study, consistent with what has been found in another similar study,^17^ and likely because targeted therapies for *EGFR* and *ALK* have been available for longer.

Patient characteristics played an important role in the likelihood of patients undergoing biomarker testing. While the demographics and clinical characteristics of the patients in our study were similar to those reported in other retrospective cohort studies,^18^, ^19^ we found that current smokers and patients with an ECOG PS of 2 or higher were less likely to be tested. Furthermore, compared with White patients, Black patients were significantly less likely to be tested. Patients in the Other Race category (American Indian or Alaska Native, Asian, Native Hawaiian or Other Pacific Islander, or other race) were significantly more likely to be tested. Other studies have found similar differences surrounding biomarker testing in patients with aNSCLC, with Asian patients being more frequently tested and Black patients less frequently tested.^20^, ^21^ *EGFR* mutations are more common in Asian patients.^22^ However, the NCCN Guidelines recommend biomarker testing for all patients, irrespective of race.^5^ Future research could assess access to molecular testing, including the relationship between health insurance type and biomarker testing rates.

During the entire observational period, chemotherapy was found to be the most common treatment class for all three LoTs in our study. This is different from another real‐world study that found chemotherapy was most commonly used in 1L and immunotherapy was most commonly used in 2L and 3L.^23^ However, when assessed from 2016 through 2020, treatment patterns in our study changed, and most patients received a targeted treatment. Our study period likely contributed to chemotherapy being identified as the most common treatment class. Despite this, only 55.7% of patients with positive biomarkers received NCCN‐recommended treatment options.

The results of our study highlight the importance of adherence to NCCN‐recommended treatment options. Patients with aNSCLC and positive biomarkers who received NCCN‐recommended treatment options had significantly longer median OS and rwPFS than those treated with non‐recommended regimens. Ideally, biomarker testing is recommended prior to treatment initiation.^24^ However, studies have shown that many patients do not have results prior to initiation of 1L treatment. One study found that only 35% of patients had biomarker testing results for *EGFR*, *ALK*, *ROS1*, *BRAF*, and PD‐L1 available before 1L treatment initiation.^13^ The results of our study suggest that adherence to NCCN Guidelines (i.e., targeted treatment determined by biomarker testing) improves real‐world clinical outcomes among patients with positive biomarkers and aNSCLC.

### 4.1 Limitations

Limitations of real‐world databases apply to this study. The rate of biomarker testing could be underestimated as testing, especially negative results, might not be recorded and would not be observed during EHR data abstraction. EHR data sources suffer from missing or incomplete follow‐up, which may introduce bias. By using an EHR data source in conjunction with case report forms, these biases were expected to be mitigated. Additionally, we could not assess trends in different types of testing over time due to the nature of the TEMPUS database. As the MM and CG TEMPUS modalities only contain patients who received biomarker testing, results related to patterns of biomarker testing could only be assessed using the CO modality. Furthermore, in the CO modality, there was a decrease in biomarker testing after 2018, so the sample size was not large enough to assess trends in specific biomarkers over time. Additionally, the NCCN Guidelines have changed over the time of the study, and not all actionable mutations in 2021 were present in the NCCN Guidelines between 2012 and 2018.

LoTs were defined by a set of business rules applied to the database and are likely to differ from those in the clinical setting. Exposure and outcome misclassifications may be present.

There are also limitations associated with survival outcomes. It is possible that patients in this study received the diagnosis of aNSCLC prior to the date of initial diagnosis observed in the database or that progression could lead to patients seeking care outside the participating institutions. Either of these could result in an underestimation of the survival outcomes. Survival outcomes are subject to survivorship bias and temporal selection bias. For outcomes such as PFS, restricting analyses to treatment post‐sequencing may bias the cohort toward patients who are considered to be high risk. The timing of biomarker testing may be associated with progressive cancer, leading to immortal time bias or left truncation of observable events.

The generalizability of the results of this study to all patients with aNSCLC in the US and beyond is limited because the TEMPUS network includes only patients seeking care with participating providers and institutions.

## 5 CONCLUSION

Despite an increase in targeted therapies for aNSCLC and ease of biomarker testing, fewer than half of the aNSCLC patients identified in the TEMPUS dataset were tested for biomarkers. Men, Black patients, current smokers, patients with squamous aNSCLC, and patients with an ECOG PS of 2+ were less likely to be tested than patients in the reference groups for each characteristic. Patients with aNSCLC and positive biomarkers who received NCCN‐recommended treatment options had significantly longer median OS and PFS than those treated with non‐recommended regimens. Adherence to NCCN Guidelines (i.e., targeted treatments) improves real‐world clinical outcomes among patients with aNSCLC and positive biomarkers.

## AUTHOR CONTRIBUTIONS

**Mo Yang:** Conceptualization (equal); investigation (equal); methodology (equal); software (equal); validation; formal analysis (equal); resources; data curation; visualization (equal), supervision, project administration, funding acquisition; writing ‐ original draft (equal); writing – review and editing (equal). **Joanna P. MacEwan:** Formal analysis (equal); software (equal); investigation (equal); methodology (equal); visualization (equal) writing – original draft (equal); writing – review and editing (equal). **Sai Sriteja Boppudi:** Writing – review and editing (equal). **Monica R. McClain:** Writing – review and editing (equal). **Richard M. O'Hara Jr:** Conceptualization (equal); writing – review and editing (equal). **Paul K. Paik:** Writing – review and editing (equal).

## FUNDING INFORMATION

This study was funded by EMD Serono (CrossRef Funder ID: 10.13039/100004755).

## CONFLICT OF INTEREST STATEMENT

MY: Employee of EMD Serono, Rockland, MA, USA; JPME: Employee of Genesis Research Group; SSB: Employee of Genesis Research Group; MRMC: Employee of Genesis Research Group at the time of the study. RMOH: Employee of EMD Serono, Rockland, MA, USA; PKP: reported advisory board funds, institutional research funding, and/or personal fees from EMD Serono, Takeda DSMC, Janssen, Xencor, Boehringer Ingelheim, CrownBio, Mirati, Calithera, and Novartis.

## Supporting information


Table S1.

## Data Availability

Any requests for data by qualified scientific and medical researchers for legitimate research purposes will be subject to the healthcare business of Merck KGaA, Darmstadt, Germany’s (CrossRef Funder ID: 10.13039/100004755) Data Sharing Policy. All requests should be submitted in writing to the healthcare business of Merck KGaA, Darmstadt, Germany’s data sharing portal (https://www.emdgroup.com/en/research/our‐approach‐to‐research‐and‐development/healthcare/clinical‐trials/commitment‐responsible‐data‐sharing.html). When the healthcare business of Merck KGaA, Darmstadt, Germany has a co‐research, co development, or co‐marketing or co‐promotion agreement, or when the product has been out‐licensed, the responsibility for disclosure might be dependent on the agreement between parties. Under these circumstances, the healthcare business of Merck KGaA, Darmstadt, Germany will endeavor to gain agreement to share data in response to requests.
