# Supplementary material for: Diagnosis, testing, treatment, and outcomes among patients with advanced non‐small cell lung cancer in the United States
Source: Cancer Med. 2023 Dec 7;12(24):21605–14. doi: 10.1002/cam4.6694 (PMC10757108; doi:10.1002/cam4.6694)
Supplement: Supplementary file 1 — Table S1. [file CAM4-12-21605-s001.docx]

**Supplemental Table (Online Only). Treatment Patterns and Duration of Treatment Among Treated Patients with Advanced Non-Small Cell Lung Cancer, by Line of Treatment and Drug Class^a^**

|  | **LoT 1** | | **LoT 2** | | **LoT 3** | |
| --- | --- | --- | --- | --- | --- | --- |
| **Drug class** | **No. (%)** | **Median (IQR) DoT, days** | **No. (%)** | **Median (IQR) DoT, days** | **No. (%)** | **Median (IQR) DoT, days** |
| **2012–2022** |  |  |  |  |  |  |
| n | 6,391 |  | 2,771 |  | 1,085 |  |
| Chemotherapy | 3,598 (56.3) | 83 (43-144) | 1,202 (43.4) | 74 (42-139) | 558 (51.4) | 84 (42-156) |
| Chemotherapy + CPI | 1,265 (19.8) | 183 (98-356) | 192 (6.9) | 144 (64-266) | 70 (6.5) | 156 (80-280) |
| CPI | 766 (12.0) | 135 (58-320) | 908 (32.8) | 112 (50-267) | 254 (23.4) | 106 (50-320) |
| TKI | 699 (10.9) | 251 (92-474) | 408 (14.7) | 157 (76-371) | 166 (15.3) | 102 (54-227) |
| Other | 63 (1.0) | N/A | 61 (2.2) | N/A | 37 (3.4) | N/A |
| **2016–2020** |  |  |  |  |  |  |
| n | 4,410 |  | 1,655 |  | 586 | |
| Chemotherapy | 1,650 (37.4) |  | 559 (33.8) |  | 295 (50.3) |  |
| Chemotherapy + CPI | 1,234 (28.0) |  | 156 (9.4) |  | 55 (9.4) |  |
| CPI | 932 (21.1) |  | 665 (40.2) |  | 129 (22.0) |  |
| TKI | 538 (12.2) |  | 242 (14.6) |  | 77 (13.1) |  |
| Other | 56 (1.3) |  | 33 (2.0) |  | 30 (5.1) |  |

**Abbreviations:** CPI, checkpoint inhibitor; DoT, duration of treatment; IQR, interquartile range; LoT, line of treatment; N/A, not applicable;

TKI, tyrosine kinase inhibitor

^a^Percentages may not equal 100% due to rounding.
